# The Association between Marital Status and Obesity: A Systematic Review and Meta-Analysis

**DOI:** 10.3390/diseases12070146

**Published:** 2024-07-05

**Authors:** Tamara Nikolic Turnic, Vladimir Jakovljevic, Zulfiya Strizhkova, Nikita Polukhin, Dmitry Ryaboy, Mariia Kartashova, Margarita Korenkova, Valeriia Kolchina, Vladimir Reshetnikov

**Affiliations:** 1 Department of Pharmacy, Faculty of Medical Sciences, University of Kragujevac, 34000 Kragujevac, Serbia; 2 N.A. Semashko Public Health and Healthcare Department, F.F. Erismann Institute of Public Health, I.M. Sechenov First Moscow State Medical University, 119435 Moscow, Russia; strizhkova_z_a@staff.sechenov.ru (Z.S.); dmr301099@gmail.com (D.R.); maria.a001mp77@gmail.com (M.K.); rita_korenkova@mail.ru (M.K.); slera25@bk.ru (V.K.); reshetnikov_v_a@staff.sechenov.ru (V.R.); 3 Department of Physiology, Faculty of Medical Sciences, University of Kragujevac, 34000 Kragujevac, Serbia; drvladakgbg@yahoo.com; 4 1st Moscow State Medical, Department of Human Pathology, University IM Sechenov, Trubetskaya Street 8, Str. 2, 119991 Moscow, Russia; 5 Department of Public Health and Medical Social Sciences, Synergy University, Leningradskiy Prospect 80k46, 125315 Moscow, Russia; nikitasketch@gmail.com

**Keywords:** marital status, obesity, risk, body mass index, meta-analysis

## Abstract

Background: Obesity was included in the International Classification of Diseases in 1990 as a chronic disease characterized by the excessive accumulation of body fat and a body mass index (BMI) greater than 30 kg/m^2^. Aim: This systematic review was aimed to examine the role of marital status in determining body mass index and the risk of obesity. Methods: We performed a systematic literature search using three databases (PubMed (Medline), Embase, and Google Scholar) with the search query. Results: Of the 105 studies included in the systematic review, 76 studies (72%) reported a greater risk of obesity in married individuals compared to unmarried individuals. A meta-analysis of 24 studies included a total population of 369,499 participants: 257,257 married individuals (40,896 of whom had obesity) and 112,242 comparison subjects (single, divorced, or widowed individuals, 15,084 of whom had obesity). Odds ratios for obesity found a significant pooled odds ratio for obesity in married individuals compared with controls (OR 1.70; 95% CI 1.38–2.10). The socioeconomic environment was not the same throughout the period of studies analyzed. The odds of obesity in married individuals during economic crises was greater than during the period between crises: OR 2.56 (95% CI 2.09–3.13) during crises vs. OR 1.55 (95% CI 1.24–1.95) between crises. Conclusion: The results of this review confirm the importance of considering marital status in determining the risk of obesity.

## 1. Introduction

Over the past years, both in developed and developing countries, there has been a sudden increase in the number of people suffering from obesity and related chronic conditions. Obesity constitutes a risk factor for diseases such as hypertension, type 2 diabetes, and cancer [1,2]. Obesity was included in the International Classification of Diseases (ICD-10) in 1990 as a chronic disease characterized by the excessive accumulation of body fat and a body mass index (BMI) greater than 30 kg/m^2^. The World Health Organization (WHO) reported that the worldwide prevalence of the obesity has tripled since 1975 [3].

It is now well established from a variety of studies that obesity is a complex disease with several known risk factors, including low physical activity, unbalanced diet, endocrine diseases, genetic predisposition, household wealth, and occupation. Marital status has been demonstrated to be an important social factor. Much of the literature emphasizes that being married is associated with a lower risk of non-communicable morbidity and mortality. This indicates a need to understand the various perceptions of marriage as a protective or risk factor that exist among literature.

Aizer, A. et al. (2013) revealed a substantially greater risk of metastatic cancer and mortality from 10 major cancer sites in unmarried compared to married individuals [4]. The results of the meta-analysis conducted by Krajc, K. et al. (2022) were in line with Aizer’s findings, reporting better overall and cancer-specific survival in married compared to unmarried patients [5].

Wong, C. et al. (2018) performed a systematic review and meta-analysis that included 34 studies from different nations and found that marriage was related to decreased cardiovascular morbidity and mortality. Unmarried participants were 1.4 times more likely to develop cardiovascular disease and die of cardiovascular diseases and stroke than married people [6].

Sommerlad, A. et al. (2018) discovered in their meta-analysis that lifelong single individuals had a 42% greater risk of having dementia and widowed individuals had a 20% greater risk of having dementia compared to married individuals [7].

The meta-analysis of Wang, Y. et al. (2020), which comprised 21 prospective cohort studies with a total of 7,881,040 individuals and 1,888,752 deaths, concluded that being unmarried conferred a higher risk of all-cause, cancer, cardiovascular disease, and coronary heart disease mortalities for both sexes [8].

However, studies in recent decades have found that marriage is associated with changes in BMI and behaviors that contribute to obesity. The relationship between marital status and obesity can be attributed to a number of factors, including changes in lifestyle, eating habits, and social support. When individuals enter into marriage, they often experience a shift in their daily routines, which can impact their health behaviors.

Dinour, L. et al. (2011) conducted a systematic review consisting of 20 studies on BMI results before and after marriage. The data for these articles were gathered over a 40-year period, from 1966 to 2004. According to Dinour’s results, marriages were associated with an increase in body weight, whereas divorces were associated with a decrease in body weight, both in males and females [9]. However, the relatively small sample size of the available research limited the scope of this review. Sixteen of the 20 studies included in the review were carried out in the United States. Furthermore, the review did not include a quantitative analysis. 

The studies presented thus far provide evidence that marital status is associated with both better health outcomes for many non-communicable diseases, but a negative impact on BMI and the risk of obesity which is considered to be a risk factors for such diseases. These contradictory data require in-depth study and prompted the first meta-analysis of the association between marital status and the risk of obesity. The aim of the study was to investigate the association between marital status and the risk of obesity based on the meta-analysis.

## 2. Materials and Methods

The study protocol was registered in the international prospective register of systematic reviews (PROSPERO) under number CRD42021292440. The preferred reporting items for systematic reviews and meta-analyses (PRISMA) statement was used as a guide to write this review.

### 2.1. Search Methods

We performed a systematic literature search using three databases (PubMed (Medline), Embase, and Google Scholar) with the search query: (‘marital status’ OR ‘marri*’ OR ‘family status’) AND (‘obesity’ OR ‘adiposity’) AND (‘body mass’ OR ‘body mass index’ OR ‘bmi’ OR ‘anthropometry’) AND (‘risk’ OR ‘prevalence’) AND ([adult]/lim OR [aged]/lim OR [very elderly]/lim) AND ‘article’/it.

A comprehensive literature search was conducted during the time period from 5 December 2021 to 4 February 2022. No language or publication date restrictions were applied. This ensured the inclusion of relevant research and allowed for an effective and integrated approach to addressing the research question. The authors of the relevant articles were contacted to obtain the full texts and additional information of the articles if they were not available.

### 2.2. Selection Criteria

We included observational studies evaluating the association between marital status and obesity. We applied no restrictions on the region of the study population. Abstracts and studies not reporting numerical data were excluded. We also excluded studies reporting overweight BMI thresholds of 25–29 kg/m^2^ or lower for Asian populations. All of the studies that analyzed the BMI as a numerical outcome, using averages, were also excluded. The eligibility of the full texts of articles was assessed by two investigators independently. Disagreements were discussed and, if needed, resolved by a third reviewer not involved in the initial selection.

The PICO (population, intervention, comparison, outcome) format was applied to formulate a focus question and to develop a systematic search strategy for the study, accordingly. Population: Adults worldwide. We excluded studies investigating obesity in pregnant women and adolescents younger than 15 years old. Intervention: No intervention considered. Comparison: Comparison subjects were married and unmarried (single, divorced, widowed) individuals. Outcome: Obesity based on the BMI. The BMI threshold for obesity varies with ethnicity. Researchers attribute this to ethnic characteristics, including average height, muscle mass, and fat mass. Asians have been found to express signs of obesity with lower BMI values on average compared to Europeans. The WHO recommends the use of lower BMI levels for obesity in Asia-Pacific countries (BMI ≥ 28 kg/m^2^ or BMI ≥ 25 kg/m^2^) [10]; thus, the outcome was considered eligible for the studies on respective populations.

### 2.3. Data Extraction and Quality Assessment

From each of the selected articles, we obtained the following data: first author; year of publication; country; study design and duration; sample size; participants characteristics, including their sex; age; marital status; group sizes; odds ratio (OR) and 95% confidence interval (95% CI).

The data extraction and the risk of bias were first assessed within the selected studies independently by two investigators, disagreements were discussed and, if needed, resolved by a third reviewer. We used the Newcastle–Ottawa scale adapted for cross-sectional studies to assess the risk of systematic error in the included studies.

### 2.4. Data Synthesis and Analysis

The analysis was performed in the software program Review Manager 5.4.1. Data synthesis was based on the recommendations of the Cochrane collaboration. Pooled odds ratios and 95% confidence intervals were calculated for primary binary data obtained from included studies. 

We used Cochrane’s Q test and the I2 index to estimate the heterogeneity of effect sizes. Since heterogeneity was high and significant in conducted meta- and submeta-analyses, DerSimonian and Laird’s random random effects model was chosen to synthesize the results from selected studies.

## 3. Results

**Figure 1:**
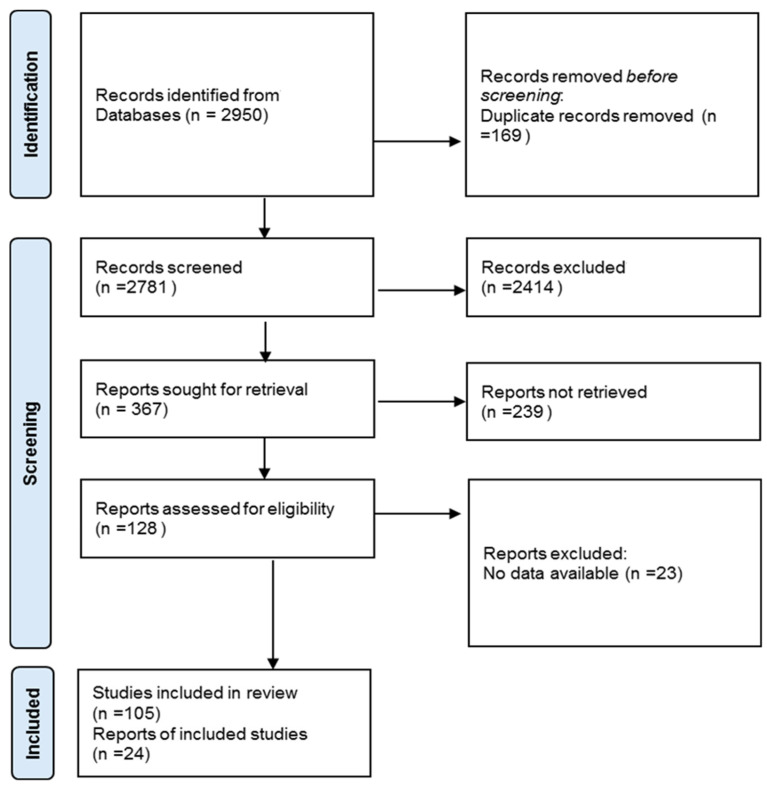
The flow chart for screening and selection of articles according to PRISMA statement.

As a result of database screening, 2950 publications were received, which were checked for compliance with the inclusion criteria. After the removal of duplicates and exclusion of non-eligible articles, 128 articles were included for further eligibility assessment. One hundred and five articles provided the data eligible for analysis and were therefore evaluated in this systematic review (Figure 1).

The selected studies were published between 1991 and 2021. The age of participants in the eligible studies lied within a range between 15 and 87 years. All 105 selected studies adopted multivariate logistic regression to investigate the association between obesity and multiple social factors, including marital status as one of the independent variables [11,12,13,14,15,16,17,18,19,20,21,22,23,24,25,26,27,28,29,30,31,32,33,34,35,36,37,38,39,40,41,42,43,44,45,46,47,48,49,50,51,52,53,54,55,56,57,58,59,60,61,62,63,64,65,66,67,68,69,70,71,72,73,74,75,76,77,78,79,80,81,82,83,84,85,86,87,88,89,90,91,92,93,94,95,96,97,98,99,100,101,102,103,104,105,106,107,108,109,110,111,112,113,114,115,116,117]. Out of 105 eligible studies, 29 used nationwide data, including Asian and African regions. Table 1 presents the most extensive national studies that included an assessment of the relationship between BMI and various social factors. 

Of the 105 studies included in the systematic review, 76 studies (72%) reported a greater risk of obesity in married individuals compared to unmarried individuals. We retrieved and examined covariate-adjusted odds ratios for the evaluation of the association between marital status and obesity. We identified significant results, provided as odds ratio and 95% confidence intervals, in 20 papers.

The selected studies provided controversial findings for obesity in sex groups. Some studies showed that women have a greater risk of obesity, while others stated the opposite. It should be noted that 7 of the 105 studies were performed solely on female populations. Separate odds ratio results for men were reported in 20 articles. Ten of them reported a significantly higher risk of obesity in married men, while in the other 10, the association between marital status and the risk of obesity in men was not significant. Regarding women, separate odds ratios for this group were published in 22 studies. Thirteen showed a significantly greater risk of obesity for married women, eight studies reported no significant differences between married and unmarried women, and one study reported a significant result indicating that the risk of obesity was higher in unmarried women.

Of particular interest for this systematic review’s focus was the study conducted in China in 2011–2012 on a sample of 10,448 pairs of same-sex twins aged 18–79 years. The findings indicated that marital status and BMI were associated regardless of genetic and common environmental factors in both sexes. Married twins had a higher BMI and a higher risk of overweight and obesity in both sex groups compared to unmarried twins [66].

**Figure 2:**
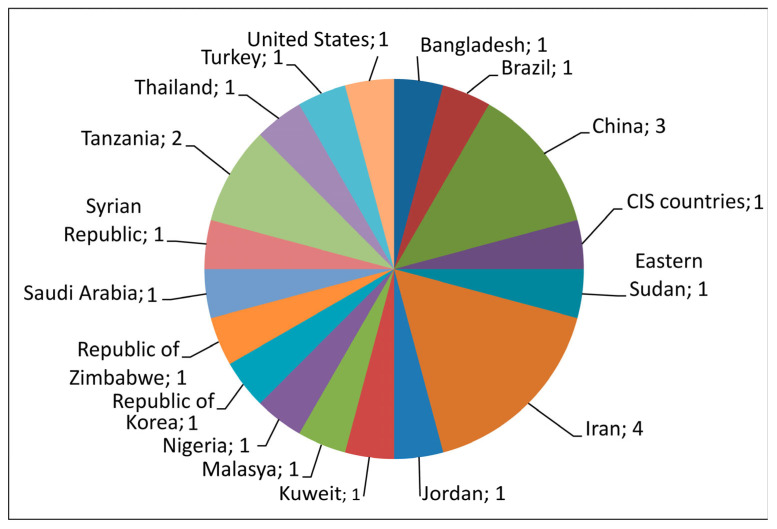
Geographical locations of the 24 studies included in the meta-analysis.

A total of 24 studies were selected for meta-analysis based on the availability of primary data on the number of participants with obesity and normal weight in groups of married and unmarried (single, divorced, widowed) individuals that were compared. The geographical locations of the selected studies covered 18 countries (Figure 2). 

**Figure 3:**
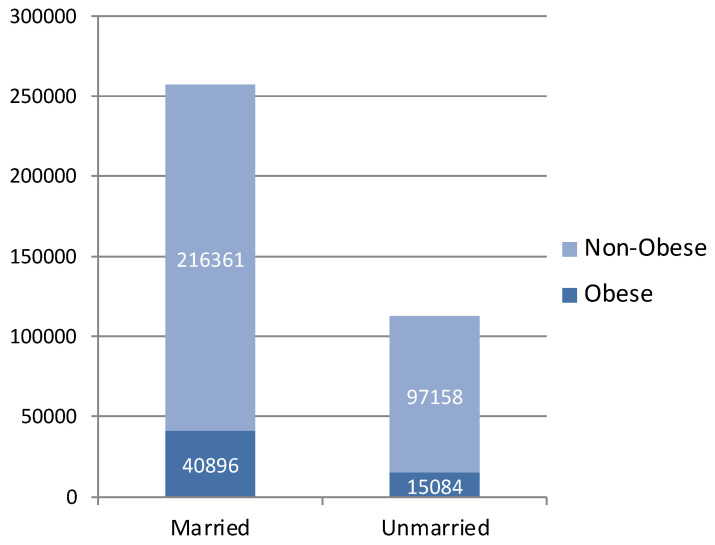
The proportions of participants having obesity in groups of married and unmarried (single, divorced, widowed) individuals.

The 24 studies with data that could be pooled for meta-analysis included 55,980 cases (15.15%) of obesity among 369,499 individuals. The proportions of individuals with obesity and individuals with normal weight in married and unmarried participants are shown in Figure 3.

Table 2 shows the summary of the characteristics and key findings of the studies included in the meta-analysis. Twenty-two of them were cross-sectional studies, and two were cohort studies that included a survey, implying that they also adopted a cross-sectional design to gather data. Similarly designed studies selected for meta-analysis produced results that were comparable.

**Figure 4:**
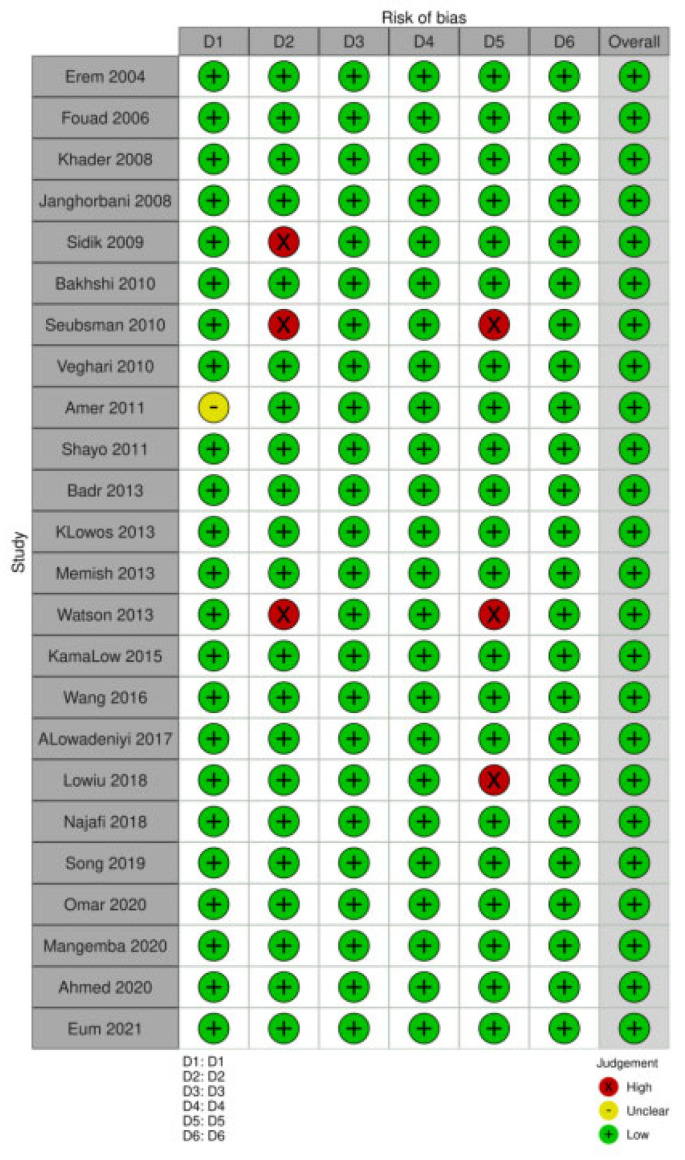
Details of the Newcastle–Ottawa scale score for studies included in meta-analysis.

On assessing study quality using the Newcastle–Ottawa scale modified for cross-sectional studies, we found that all 24 studies selected for the meta-analysis were deemed to be of high quality (7–9 score), thus providing a low risk of bias (Figure 4).

Of the 24 studies, 19 showed a significant association between marital status and obesity. Eighteen studies reported that married individuals had a greater risk of obesity than unmarried individuals. A meta-analysis of 24 studies included a total population of 369,499 participants: 257,257 married individuals (40,896 of whom had obesity) and 112,242 comparison subjects (single, divorced, or widowed individuals, 15,084 of whom had obesity). 

**Figure 5:**
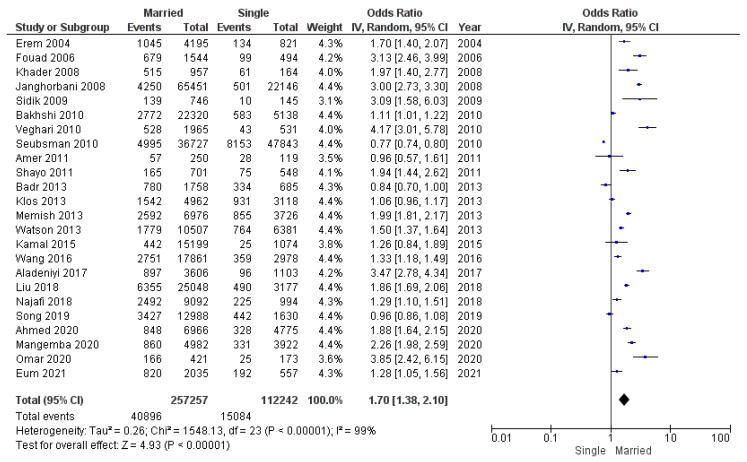
Meta-analysis of odds ratios and 95% confidence intervals for marital status and obesity.

Our first analysis of all available studies reporting odds ratios for obesity found a significant pooled odds ratio for obesity in married individuals compared with controls (OR 1.70; 95% CI 1.38–2.10) (Figure 5). Heterogeneity was high and significant. This may be attributed to the differing sample sizes employed in the included studies, as well as the ethnic characteristics that may have influenced the predisposition to obesity. The observed heterogeneity in the selected studies may also be attributed to the varying age compositions of the study populations, as age-related patterns in BMI have been identified.

Considering differences in obesity BMI thresholds, we conducted submeta-analyses of the respective subgroup of studies: BMI ≥ 30 kg/m^2^, BMI ≥ 28 kg/m^2^, and BMI ≥ 25 kg/m^2^.

**Figure 6:**
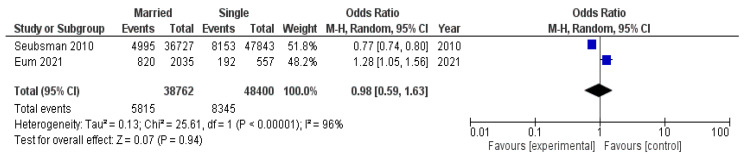
Submeta-analysis of odds ratios and 95% confidence intervals for marital status and obesity in a group of studies with an obesity threshold of BMI ≥ 25 kg/m^2^.

A submeta-analysis of two studies with an obesity threshold of BMI ≥ 25 kg/m^2^ included a total population of 87,162 participants (14,160 of whom had obesity): 38,762 married individuals (5815 of whom had obesity) and 48,400 unmarried individuals (8345 of whom had obesity). As shown in Figure 6, the pooled odds ratio in this group of studies was not significant (ОR 0.98, 95% CI 0.59–1.63). Heterogeneity was high and significant.

**Figure 7:**
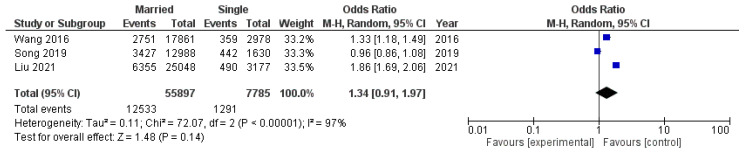
Submeta-analysis of odds ratios and 95% confidence intervals for marital status and obesity in a group of studies with an obesity threshold of BMI ≥ 28 kg/m^2^.

A submeta-analysis of three studies with an obesity threshold of BMI ≥ 28 kg/m^2^ included a total population of 63,682 participants (13,824 of whom had obesity): 55,897 married individuals (12,533 of whom had obesity) and 7785 unmarried individuals (1291 of whom had obesity). From this data (Figure 7), we can see that the group resulted in a greater but still not significant pooled odds ratio (ОR 1.34, 95% CI 0.91–1.97). Heterogeneity was high and significant.

**Figure 8:**
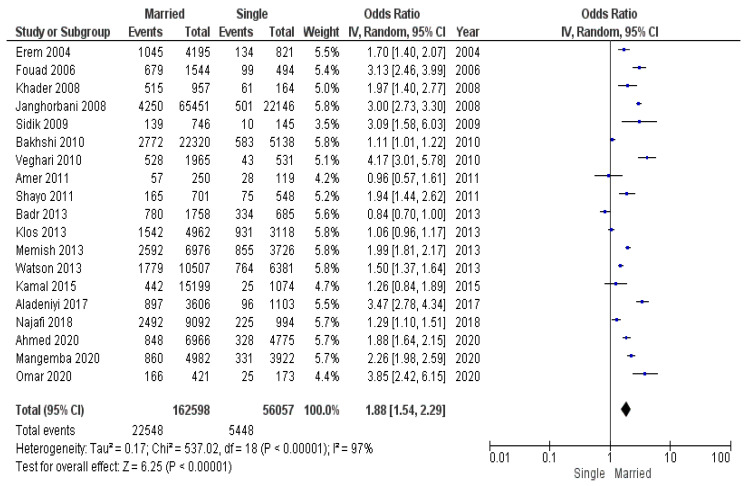
Submeta-analysis of odds ratios and 95% confidence intervals for marital status and obesity in a group of studies with an obesity threshold of BMI ≥ 30 kg/m^2^.

The last subgroup of studies that had an obesity threshold of BMI ≥ 30 kg/m^2^ included a total population of 218,655 participants (27,996 of whom had obesity): 162,598 married individuals (22,548 of whom had obesity) and 56,057 unmarried individuals (5448 of whom had obesity). The submeta-analysis of this group showed that the exclusion of studies with a lower obesity BMI threshold resulted in a greater odds ratio (OR 1.88, 95% CI 1.54–2.29) compared to the meta-analysis of all 24 studies (Figure 8). Heterogeneity was high and significant.

Odds ratios for meta-analysis of all included studies and submeta-analyses are presented in Table 3. The most interesting conclusion drawn from the figures provided in a table is that there is a considerable influence of marital status on the odds of obesity among married Europeans, because both the criteria for obesity are higher for them and the pooled odds ratio in the respective meta-analysis was greater than in the meta-analysis that included studies from all populations.

**Figure 9:**
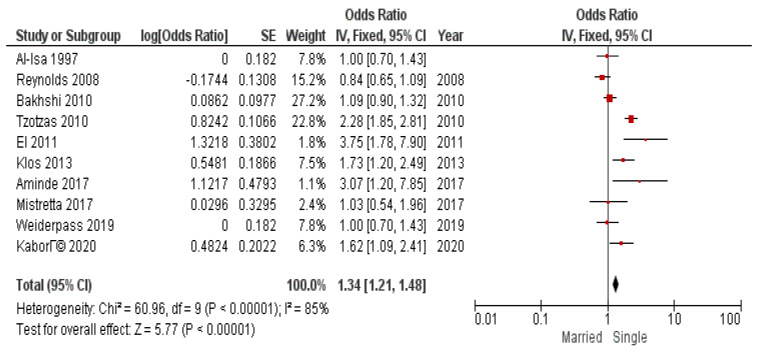
Submeta-analysis of odds ratios and 95% confidence intervals for marital status and obesity in a subgroup of women.

**Figure 10:**
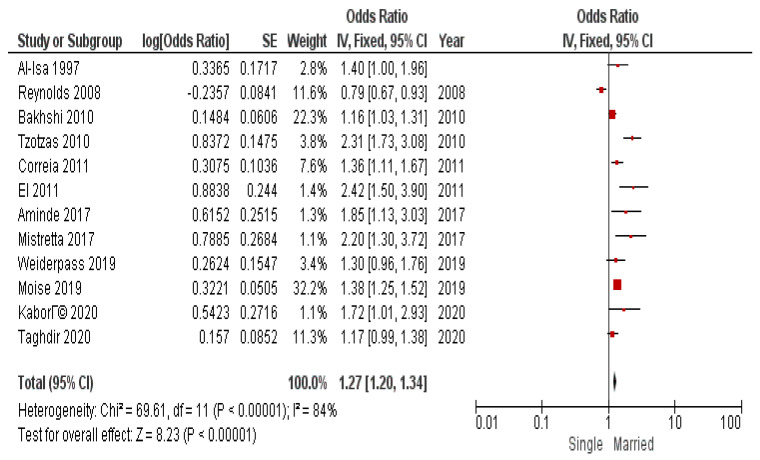
Submeta-analysis of odds ratios and 95% confidence intervals for marital status and obesity in a subgroup of men.

In addition to BMI threshold division, it was considered to conduct submeta-analyses to investigate the association of marital status and obesity in sex subgroups. The results of submeta-analyses in sex subgroups are presented in Figure 9 and Figure 10. 

We obtained significant pooled odds ratio for both subgroups: OR 1.34 (95% CI 1.21–1.48) for men and OR 1.27 (95% CI 1.20–1.34) for women. Heterogeneity was high and significant.

**Figure 11:**
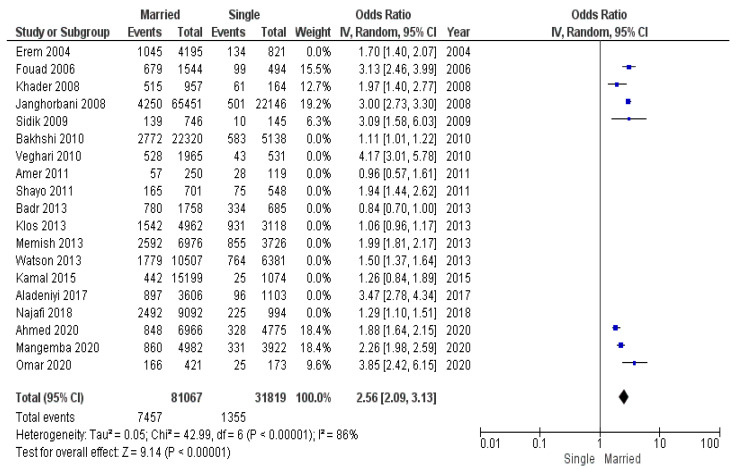
Submeta-analysis of odds ratios and 95% confidence intervals for marital status and obesity in a studies conducted during economic crises of 2008–2009 and 2020–2021.

**Figure 12:**
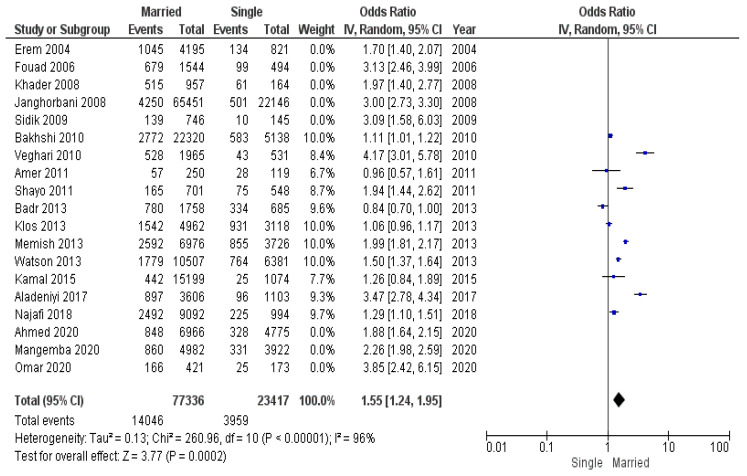
Submeta-analysis of odds ratios and 95% confidence intervals for marital status and obesity in a studies conducted between economic crises from 2010 to 2019.

The socioeconomic environment was not the same throughout the period of studies analyzed. We made the assumption that, during a crisis and decreased socioeconomic conditions, fewer people have the ability to maintain healthy lifestyle and purchase quality food. Considering this fact, we conducted a submeta-analysis of subgroups: (1) studies conducted during the global economic crises of 2008–2009 and 2020–2021 (Figure 11), (2) studies conducted between 2010 and 2019 (Figure 12).

The group of studies conducted during the economic disturbances of 2008–2009 and 2020–2021 consisted of 112,886 participants (8812 of whom had obesity): 81,067 married individuals (7457 of whom had obesity) and 31,819 unmarried individuals (1355 of whom had obesity). 

The group of studies conducted between economic crises consisted of 100,753 participants (18,005 of whom had obesity): 77,336 married individuals (14,046 of whom had obesity) and 23,417 unmarried individuals (3959 of whom had obesity). 

According to pooled odds ratio for these subgroups, the odds of obesity in married individuals during economic crises was greater than during the period between crises: OR 2.56 (95% CI 2.09–3.13) during crises vs. OR 1.55 (95% CI 1.24–1.95) between crises.

## 4. Discussion

Despite a multiplicity of obesity prevention strategies in both developed and developing countries, the prevalence of obesity continues to rise. The rapid expansion in the prevalence of obesity-related diseases is imposing a substantial burden on healthcare systems worldwide. Genetic predisposition cannot account for the accelerated increase in obesity observed in recent decades within specific ethnic groups and across the globe.

Over the past several decades, a substantial body of research has been conducted on obesity among the population. A significant proportion of these studies have focused on identifying risk factors for obesity. Among those risk factors, social factors have received considerable attention. Our study summarized all available published data and showed that married individuals have higher odds of developing obesity compared to unmarried (single, divorced, widowed) individuals. There are several hypotheses that may potentially explain our findings. 

The first hypothesis may be expressed as follows: over time, couples who share a household establish shared routines, eating habits, and preferred activities. In such circumstances, the probability of developing shared unhealthy habits increases. If one partner gains weight, the other partner is more likely to gain weight as well. Furthermore, the sense of security and peace of mind that accompany marriage may have a direct effect on metabolic rate. Adipose tissue accumulates when metabolic activities are slowed [118].

There is also the contrary hypothesis that excessive weight in one or both partners may be an indicator of issues in a couple’s relationship. The disappointment and depression may cause a so-called “stress eating” behavior [119].

One of possible factors associated with marriage is the commitment that is inherent in the formation of a family. The sharing of a household and the desire to spend time with a partner occupy a significant portion of leisure time, and if not sufficiently motivated, can overshadow health issues. In order to maintain sufficient physical activity, a person must apply additional efforts and be engaged in it regularly. This necessitates the allocation of time, financial resources, and, most crucially, willpower. The presence of children also entails an additional responsibility for the couple, with their leisure time often devoted to the care of the child.

Another hypothesis explains the association between early marriage and obesity risk. This concept is particularly applicable in developing nations, where the rate of early marriage is higher due to religious, economic, and social factors. A lower socioeconomic level in comparison to developed nations may be another factor impeding individuals from maintaining a healthy lifestyle [120,121].

These hypotheses require further investigation. However, preventive measures should be focused on improving relationships within the couple and efforts to build shared healthy habits. Partners should discover leisure activities that are both in their best interests and allow them to sustain healthy behaviors.

Taken together, the findings of the actual study empower the idea that the development of family weight loss programs can be very effective in preventing obesity. Family programs will increase the likelihood of forming new healthy habits within couples and provide support and motivation for healthy behaviors. It may be beneficial to find such activities that are mutually agreeable by both partners. It can be reasonably asserted that the prevention of disease and the maintenance of optimal health are contingent on the establishment and sustenance of a harmonious and trusting relationship between the couple. Individuals who are married or cohabiting should pay more attention to their body weight.

The findings of this study can be utilized to mitigate the adverse effects of social and environmental factors, particularly those associated with marriage and cohabitation. From a public health perspective, it is crucial to highlight the risk and preventive factors of diseases. However, the fact that obesity increases the risk of the development of hypertension, type 2 diabetes, and cancer is contrary to data showing a lower prevalence of these diseases among married people. In this regard, the hypothesis that cohabitation with a partner is a protective factor that exceeds the risks associated with obesity requires further investigation.

### Limitations

The actual study is not without limitations. A major limitation of this review is the design of the included studies. Even when undertaken as part of longitudinal investigations, the studies included in the meta-analysis were cross-sectional. However, the significant effect sizes found in several of the studies indicate a high level of quality in these research data. Another limitation is that the studies lacked information on the length of marriage, cohabitation, or singleness. This prevented the effects of duration of cohabitation or single living on obesity from being investigated. As an additional source of limitation, it is worth mentioning the possible variation in the measurements used in the studies carried out in the different countries. Future studies should explore this further.

## 5. Conclusions

The results of this review confirm the importance of considering marital status in determining the risk of obesity. The odds of obesity were 88% higher among married individuals compared with single, divorced, and widowed individuals (considering the WHO recommended obesity threshold of BMI ≥ 30 kg/m^2^). No significant differences in odds were found in Asia-Pacific countries with lower obesity BMI thresholds (BMI ≥ 25 kg/m^2^ or ≥ 28 kg/m^2^). The odds of obesity in married men did not differ from that of women.

During the 2008–2009 and 2020–2021 global economic crises, the odds of obesity in married individuals increased compared to the period between crises.

Individuals who are married or cohabiting should be advised to control their weight more strictly and take regular measurements, maintain a healthy diet, and engage in physical activity.

## Figures and Tables

**Table 1:** Summary of findings of articles based on nationally representative observational studies.

First Author, Year of Publication	Country	Period of Observation	Number of Participants	Evaluated Factors of Obesity	Results for Marital Status and Obesity
Zhang, 2020 [116]	China	2012–2015	441,306	Sex, age, education level, smoking, alcohol consumption, marital status, family history of cardiovascular disease	A significantly higher prevalence of overweight and obesity was found among married and cohabiting individuals compared to those who are unmarried, OR 1.16 (95% CI 1.07–1.25).
Gouda, 2014 [45]	India	2005–2006	124,385 women	Age, religion, caste, education level, marital status, parity, work status, region, mass media exposure	Married women were 1.86 and 2.14 times more likely to be overweight or to have obesity, respectively, than unmarried women, RR 2.14 (95% CI 1.680–2.729).
Janghorbani, 2008 [53]	Iran	2004–2005	89,404	Sex, age, marital status, education level, physical activity, smoking, area of residence	Ever-married status was associated with a significantly higher risk of overweight and obesity in both men and women. The multivariate OR of obesity was three times higher in married compared to unmarried.
Seubsman, 2010 [91]	Thailand	2005–2005	87,134	Sex, age, marital status, educational attainment, monthly personal income, household assets classified by replacement value, housing type.	Obesity was substantially less prevalent among single men and women than in those with relationships.
Gong, 2021 [44]	USA, California	2013–2014	47,970 asian-americans	Sex, age, ethnicity, household income, smoking, marital status, education level, physical activity, fast food consumption	Obesity was highly prevalent among married people.
Baik, 2018 [25]	Republic of Korea	1998–2011	42,584	Sex, age, marital status, employment status, income, smoking, alcohol consumption, sleep duration, psychological factors, diet	Being married was associated with the prevalence of obesity.
Sartorius, 2015 [89]	South Africa	2008–2012	28,247	Sex, age, living in formal urban areas, white ethnicity, being married, not exercising and/or in higher socio-economic category and/or living in households with proportionate higher spending on food (and unhealthy food options)	Marriage was identified as an important determinant of obesity, with male obesity being more strongly associated with marital status. Leaving a marriage (divorce or widowhood) was associated with a considerable reduction of risk of obesity.
Hosseini, 2020 [48]	Canada	2012–2015	28,238	Age, marital status, smoking, education, social network size (sum of all social contacts), social participation (regular social activities)	Being single, widowed, or divorced/separated was associated with worse anthropometric outcomes in women.
Tzotzas, 2010 [105]	Greece	2010–2010	17,341	Gender, age, marital status, education level	A significant association between marital status and obesity was found in both sex groups.

**Table 2:** Characteristics of studies included in the meta-analysis.

№	Reference, Year of Publication	Country	Study Design	Obesity BMI Threshold	Age	Sample Size	The Results Given in the Article
1	Bakhshi et al., 2010 [26]	Iran	Cross-sectional study	≥30 kg/m^2^	20–69	26,551	Male OR 1.09 (0.9–1.32) Female OR 1.16 (1.03–1.31)
2	Erem et al., 2004 [39]	Turkey	Cross-sectional study	≥30 kg/m^2^	20 or older	5016	Obesity prevalence: in women—29.4%, in men—16.5%
3	Klos et al., 2013 [61]	United States	Cross-sectional study	≥30 kg/m^2^	20 or older	8078	Married OR 1.73 (1.20–2.52)
4	Fouad et al., 2006 [42]	Syrian Republic	Cross-sectional study	≥30 kg/m^2^	18–65	2038	Married OR 2.62 (1.13–6.10)
5	Khader et al., 2008 [60]	Jordan	Cross-sectional study	≥30 kg/m^2^	25 or older	1121	Married OR 2.7 (1.4–5.2)
6	Sidik et al., 2009 [94]	Malasya	Cross-sectional study	≥30 kg/m^2^	20–59	891	Married OR 2.70 (1.50–5.01)
7	Janghorbani et al., 2008 [53]	Iran	Cross-sectional study	≥30 kg/m^2^	15–65	87,597	Married OR 2.53 (2.25–2.85)
8	Seubsman et al., 2010 [91]	Thailand	A survey in cohort study	≥25 kg/m^2^	15–87	85,886	Obesity prevalence: Partnered males 29.4%–36.7% Partnered females 12.2%–13.2% Single males 14.8%–15.9% Single females 8.0%–8.6%
9	Badr et al., 2013 [24]	Kuweit	Cross-sectional study	≥30 kg/m^2^	50 or older	2443	Married OR 2.29 (1.69–3.08)
10	Veghari et al., 2010 [107]	Iran	Cross-sectional study	≥30 kg/m^2^	15–65	2495	Married OR 5.95 (3.54–10.0)
11	Amer et al., 2011 [18]	Brazil	Cross-sectional study	≥30 kg/m^2^	18 or older	369	Married OR 1.6 (0.63–4.11)
12	Shayo et al., 2011 [93]	Tanzania	Cross-sectional study	≥30 kg/m^2^	18–65	1249	Married OR 1.6 (1.0–2.4)
13	Watson et al., 2013 [109]	CIS countries	Cross-sectional study	≥30 kg/m^2^	18 or older	16,944	Single OR 0.62 (0.50–0.75)
14	Kamal et al., 2015 [56]	Bangladesh	Cross-sectional study	≥30 kg/m^2^	15–49	16,273	Married OR 1.01 (0.82–1.25)
15	Wang et al., 2016 [84]	China	Cross-sectional study	≥28 kg/m^2^	18–79	20,839	Married OR 1.44 (1.19–1.74)
16	Memish et al., 2013 [68]	Saudi Arabia	Cross-sectional study	≥30 kg/m^2^	15 or older	10,702	Single OR 0.53 (0.46–0.63)
17	Eum et al., 2021 [40]	Republic of Korea	Cross-sectional study	≥25 kg/m^2^	19–60	2592	Married RR 1.78 (1.25–2.54)
18	Mangemba et al., 2020 [67]	Republic of Zimbabwe	Cross-sectional study	≥30 kg/m^2^	15–49	8904	Married OR 1.54 (1.27–1.87)
19	Ahmed et al., 2020 [63]	Tanzania	Cross-sectional study	≥30 kg/m^2^	15–49	11,741	Married RR 1.78(1.25–2.54
20	Najafi et al., 2018 [75]	Iran	A survey in cohort study	≥30 kg/m^2^	35–65	10,086	Overall prevalence of obesity 26.72%
21	Aladeniyi et al., 2017 [14]	Nigeria	Cross-sectional study	≥30 kg/m^2^	24 or older	4828	Married OR 2.1 (1.7–2.8)
22	Liu et al., 2018 [112]	China	A survey in cohort study	≥30 kg/m^2^	18–79	39,034	Single OR 0.86 (0.77–0.96)
23	Omar et al., 2020 [79]	Eastern Sudan	Cross-sectional study	≥30 kg/m^2^	20 or older	594	Married OR 4.37(2.60–7.35)
24	Song et al., 2019 [98]	China	Cross-sectional study	≥28 kg/m^2^	35–80	14,618	Married OR 1.8 (1.4–2.5)

**Table 3:** Odds ratios and 95% confidence intervals for meta- and submeta-analyses based on obesity threshold of BMI.

Studies	Sample Size (N)	Odds Ratio	95% Confidence Interval for OR
All studies (n = 24)	369,499	1.70	1.38–2.10 *
Obesity threshold of BMI ≥ 30 kg/m^2^ (n = 19)	218,655	1.88	1.54–2.29 *
Obesity threshold of BMI ≥ 28 kg/m^2^ (n = 3)	63,682	1.34	0.91–1.97
Obesity threshold of BMI ≥ 25 kg/m^2^ (n = 2)	87,162	0.98	0.59–1.63

*—significant results.

## References

[1] WHO (1998). Obesity: Preventing and Managing the Global Epidemic: Report of a WHO Consultation on Obesity, Geneva, 3–5 June 1997.

[2] Hjartåker A., Langseth H., Weiderpass E. (2008). Obesity and diabetes epidemics: Cancer repercussions. Adv. Exp. Med. Biol..

[3] World Health Organization Obesity and Overweight. https://www.who.int/news-room/fact-sheets/detail/obesity-and-overweight.

[4] Aizer A.A., Chen M.-H., McCarthy E.P., Mendu M.L., Koo S., Wilhite T.J., Graham P.L., Choueiri T.K., Hoffman K.E., Martin N.E. (2013). Marital Status and Survival in Patients with Cancer. J. Clin. Oncol..

[5] Krajc K., Miroševič Š., Sajovic J., Ketiš Z.K., Spiegel D., Drevenšek G., Drevenšek M. (2022). Marital status and survival in cancer patients: A systematic review and meta-analysis. Cancer Med..

[6] Wong C.W., Kwok C.S., Narain A., Gulati M., Mihalidou A.S., Wu P., Alasnag M., Myint P.K., A Mamas M. (2018). Marital status and risk of cardiovascular diseases: A systematic review and meta-analysis. Heart.

[7] Sommerlad A., Ruegger J., Singh-Manoux A., Lewis G., Livingston G. (2018). Research paper: Marriage and risk of dementia: Systematic review and meta-analysis of observational studies. J. Neurol. Neurosurg. Psychiatry.

[8] Wang Y., Jiao Y., Nie J., O’neil A., Huang W., Zhang L., Han J., Liu H., Zhu Y., Yu C. (2020). Sex differences in the association between marital status and the risk of cardiovascular, cancer, and all-cause mortality: A systematic review and meta-analysis of 7,881,040 individuals. Glob. Health Res. Policy.

[9] Dinour L., Leung M.M., Tripicchio G., Khan S., Yeh M.-C. (2012). The Association between Marital Transitions, Body Mass Index, and Weight: A Review of the Literature. J. Obes..

[10] (2004). WHO Expert Consultation Appropriate body-mass index for Asian populations and its implications for policy and intervention strategies. Lancet.

[11] Aballay L.R., Osella A.R., Celi A., Díaz M.d.P. (2009). Overweight and obesity: Prevalence and their association with some social characteristics in a random sample population-based study in Córdoba city, Argentina. Obes. Res. Clin. Pract..

[12] Abdeen Z., Jildeh C., Dkeideek S., Qasrawi R., Ghannam I., Al Sabbah H. (2012). Overweight and Obesity among Palestinian Adults: Analyses of the Anthropometric Data from the First National Health and Nutrition Survey (1999–2000). J. Obes..

[13] Addo P.N.O., Nyarko K.M., Sackey S.O., Akweongo P., Sarfo B. (2015). Prevalence of obesity and overweight and associated factors among financial institution workers in Accra Metropolis, Ghana: A cross sectional study. BMC Res. Notes.

[14] Aladeniyi I., Adeniyi O.V., Fawole O., Adeolu M., Ter Goon D., Ajayi A.I., Owolabi E.O. (2017). Pattern and correlates of obesity among public service workers in Ondo State, Nigeria: A cross-sectional study. South Afr. Fam. Pract..

[15] Aldossari K.K., Shubair M.M., Al-Ghamdi S., Al-Zahrani J., AlAjmi M., Alshahrani S.M., Alsalamah M., Al-Khateeb B.F., Bahkali S., El-Metwally A. (2021). The association between overweight/obesity and psychological distress: A population based cross-sectional study in Saudi Arabia. Saudi J. Biol. Sci..

[16] Al-Ghamdi S., Shubair M.M., Aldiab A., Al-Zahrani J.M., Aldossari K.K., Househ M., Nooruddin S., Razzak H.A., El-Metwally A. (2018). Prevalence of overweight and obesity based on the body mass index; a cross-sectional study in Alkharj, Saudi Arabia. Lipids Health Dis..

[17] Al-Isa A.N. (1997). Temporal Changes in Body Mass Index and Prevalence of Obesity among Kuwaiti Men. Ann. Nutr. Metab..

[18] Amer N.M., Marcon S.S., Santana R.G. (2011). Body mass index and hypertension in adult subjects in Brazil’s Midwest. Arq. Bras. Cardiol..

[19] Aminde L.N., Atem J.A., Kengne A.P., Dzudie A., Veerman J.L. (2017). Body mass index-measured adiposity and population attributability of associated factors: A population-based study from Buea, Cameroon. BMC Obes..

[20] de Andrade F.B., Junior A.d.F.C., Kitoko P.M., Batista J.E.M., de Andrade T.B. (2012). Prevalence of overweight and obesity in elderly people from Vitória-ES, Brazil. Cienc. Saude Coletiva.

[21] Asahara S.-I., Miura H., Ogawa W., Tamori Y. (2020). Sex difference in the association of obesity with personal or social background among urban residents in Japan. PLoS ONE.

[22] Asil E., Surucuoglu M.S., Cakiroglu F.P., Ucar A., Ozcelik A.O., Yilmaz M.V., Akan L.S. (2014). Factors That Affect Body Mass Index of Adults. Pak. J. Nutr..

[23] El Ati J., Traissac P., Delpeuch F., Aounallah-Skhiri H., Béji C., Eymard-Duvernay S., Bougatef S., Kolsteren P., Maire B., Ben Romdhane H. (2012). Gender Obesity Inequities Are Huge but Differ Greatly According to Environment and Socio-Economics in a North African Setting: A National Cross-Sectional Study in Tunisia. PLoS ONE.

[24] Badr H.E., Shah N.M., Shah M.A. (2012). Obesity among Kuwaitis Aged 50 Years or Older: Prevalence, Correlates, and Comorbidities. Gerontologist.

[25] Baik I. (2018). Forecasting obesity prevalence in Korean adults for the years 2020 and 2030 by the analysis of contributing factors. Nutr. Res. Pract..

[26] Bakhshi E., Mohammad K., Eshraghian M.R., Seifi B. (2010). Factors related to obesity among Iranian men: Results from the National Health Survey. Public Health Nutr.

[27] Bakhshi E., Seifi B., Biglarian A., Mohammad K. (2012). Changes in Body Mass Index across Age Groups in Iranian Women: Results from the National Health Survey. J. Nutr. Metab..

[28] Barzin M., Piri Z., Serahati S., Valizadeh M., Azizi F., Hosseinpanah F. (2018). Incidence of abdominal obesity and its risk factors among Tehranian adults. Public Health Nutr.

[29] Barzin M., Keihani S., Hosseinpanah F., Serahati S., Ghareh S., Azizi F. (2015). Rising trends of obesity and abdominal obesity in 10 years of follow-up among Tehranian adults: Tehran Lipid and Glucose Study (TLGS). Public Health Nutr.

[30] Befort C.A., Nazir N., Perri M.G. (2012). Prevalence of Obesity among Adults from Rural and Urban Areas of the United States: Findings from NHANES (2005–2008). J. Rural Health.

[31] Bell C.N., Thorpe R.J. (2019). Income and Marital Status Interact on Obesity among Black and White Men. Am. J. Men’s Health.

[32] Soares D.A., Barreto S.M. (2014). Overweight and abdominal obesity in adults in a quilombo community in Bahia State, Brazil. Cad. Saude Publica.

[33] Black J.L., Macinko J. (2010). The Changing Distribution and Determinants of Obesity in the Neighborhoods of New York City, 2003–2007. Am. J. Epidemiology.

[34] Coll J.L., Bibiloni M.d.M., Salas R., Pons A., Tur J.A. (2015). Prevalence and Related Risk Factors of Overweight and Obesity among the Adult Population in the Balearic Islands, a Mediterranean Region. Obes. Facts.

[35] Correia L.L., da Silveira D.M.I., Cavalcante A., Campos J.S., Machado M.M.T., Rocha H.A.L., da Cunha A.J.L.A., Lindsay A.C. (2011). Prevalence and determinants of obesity and overweight among reproductive age women living in the semi-arid region of Brazil. Cienc. Saude Coletiva.

[36] Dahly D.L., Gordon-Larsen P., Popkin B.M., Kaufman J.S., Adair L.S. (2010). Associations between Multiple Indicators of Socioeconomic Status and Obesity in Young Adult Filipinos Vary by Gender, Urbanicity, and Indicator Used. J. Nutr..

[37] de Moraes S.A., Humberto J.S.M., de Freitas I.C.M. (2011). Nutritional and socioeconomic status in adults living in Ribeirão Preto, SP, 2006. OBEDIARP Project. Rev. Bras. Epidemiol..

[38] El Rhazi K., Nejjari C., Zidouh A., Bakkali R., Berraho M., Gateau P.B. (2010). Prevalence of obesity and associated sociodemographic and lifestyle factors in Morocco. Public Health Nutr.

[39] Erem C., Arslan C., Hacihasanoglu A., Deger O., Topbaş M., Ukinc K., Ersöz H., Telatar M. (2004). Prevalence of Obesity and Associated Risk Factors in a Turkish Population (Trabzon City, Turkey). Obes. Res..

[40] Eum M.-J., Jung H.-S. (2021). The interplay of sleep duration, working hours, and obesity in Korean male workers: The 2010–2015 Korea National Health and Nutrition Examination Survey. PLoS ONE.

[41] Mashinya F., Alberts M., Cook I., Ntuli S. (2018). Determinants of body mass index by gender in the Dikgale Health and Demographic Surveillance System site, South Africa. Glob. Health Action.

[42] Fouad M., Rastam S., Ward K., Maziak W. (2006). Prevalence of obesity and its associated factors in Aleppo, Syria. Prev. Control..

[43] Gallus S., Odone A., Lugo A., Bosetti C., Colombo P., Zuccaro P., La Vecchia C. (2012). Overweight and obesity prevalence and determinants in Italy: An update to 2010. Eur. J. Nutr..

[44] Gong S., Wang K., Li Y., Zhou Z., Alamian A. (2021). Ethnic group differences in obesity in Asian Americans in California, 2013–2014. BMC Public Health.

[45] Gouda J., Prusty R.K. (2014). Overweight and obesity among women by economic stratum in urban india. J. Health Popul. Nutr..

[46] Hajek A., Brettschneider C., van der Leeden C., Lühmann D., Oey A., Wiese B., Weyerer S., Werle J., Fuchs A., Pentzek M. (2020). Prevalence and factors associated with obesity among the oldest old. Arch. Gerontol. Geriatr..

[47] Sarma H., Saquib N., Hasan M., Saquib J., Rahman A.S., Khan J.R., Uddin J., Cullen M.R., Ahmed T. (2016). Determinants of overweight or obesity among ever-married adult women in Bangladesh. BMC Obes..

[48] Hosseini Z., Veenstra G., Khan N.A., Conklin A.I. (2020). Associations between social connections, their interactions, and obesity differ by gender: A population-based, cross-sectional analysis of the Canadian Longitudinal Study on Aging. PLoS ONE.

[49] Hosseinpanah F., Mirbolouk M., Mossadeghkhah A., Barzin M., Serahati S., Delshad H., Azizi F. (2015). Incidence and potential risk factors of obesity among Tehranian adults. Prev. Med..

[50] Hruby A., Hill O.T., Bulathsinhala L., McKinnon C.J., Montain S.J., Young A.J., Smith T.J. (2015). Trends in overweight and obesity in soldiers entering the USArmy, 1989-2012. Obesity.

[51] Ajayi I.O., Adebamowo C., Adami H.-O., Dalal S., Diamond M.B., Bajunirwe F., Guwatudde D., Njelekela M., Nankya-Mutyoba J., Chiwanga F.S. (2016). Urban–rural and geographic differences in overweight and obesity in four sub-Saharan African adult populations: A multi-country cross-sectional study. BMC Public Health.

[52] Liu J., Garstka M.A., Chai Z., Chen Y., Lipkova V., Cooper M.E., Mokoena K.K., Wang Y., Zhang L. (2021). Marriage contributes to higher obesity risk in China: Findings from the China Health and Nutrition Survey. Ann. Transl. Med..

[53] Janghorbani M., Amini M., Rezvanian H., Gouya M.-M., Delavari A., Alikhani S., Mahdavi A. (2008). Association of body mass index and abdominal obesity with marital status in adults. Arch. Iran. Med..

[54] Kaboré S., Millogo T., Soubeiga J.K., Lanou H., Bicaba B., Kouanda S. (2020). Prevalence and risk factors for overweight and obesity: A cross-sectional countrywide study in Burkina Faso. BMJ Open.

[55] Kahn H.S., Williamson D.F., A Stevens J. (1991). Race and weight change in US women: The roles of socioeconomic and marital status. Am. J. Public Health.

[56] Kamal S.M., Hassan C.H., Alam G.M. (2015). Dual Burden of Underweight and Overweight among Women in Bangladesh: Patterns, Prevalence, and Sociodemographic Correlates. J. Health Popul. Nutr..

[57] Kaplan M.S., Huguet N., Newsom J.T., McFarland B.H., Lindsay J. (2003). Prevalence and Correlates of Overweight and Obesity among Older Adults: Findings from the Canadian National Population Health Survey. J. Gerontol. Ser. A.

[58] Kee C.C., Jamaiyah H., Safiza M.N.N., Khor G.L., Suzana S., Jamalludin A.R., Rahmah R., Ahmad A.Z., Ruzita A.T., Wong N.F. (2008). Abdominal Obesity in Malaysian Adults: National Health and Morbidity Survey III (NHMS III, 2006). Malays. J. Nutr..

[59] Khabazkhoob M., Emamian M.H., Hashemi H., Shariati M., Fotouhi A. (2017). Prevalence of Overweight and Obesity in the Middle-age Population: A Priority for the Health System. Iran. J. Public Health.

[60] Khader Y., Batieha A., Ajlouni H., El-Khateeb M., Ajlouni K. (2008). Obesity in Jordan: Prevalence, Associated Factors, Comorbidities, and Change in Prevalence over Ten Years. Metab. Syndr. Relat. Disord..

[61] Klos L.A., Sobal J. (2013). Marital status and body weight, weight perception, and weight management among U.S. adults. Eat. Behav..

[62] Kowalkowska J., Poínhos R., Franchini B., Afonso C., Correia F., Pinhão S., de Almeida M.D.V., Rodrigues S. (2015). General and abdominal adiposity in a representative sample of Portuguese adults: Dependency of measures and socio-demographic factors’ influence. Br. J. Nutr..

[63] Ahmed K.Y., Rwabilimbo A.G., Abrha S., Page A., Arora A., Tadese F., Beyene T.Y., Seiko A., Endris A.A., Agho K.E. (2020). Factors associated with underweight, overweight, and obesity in reproductive age Tanzanian women. PLoS ONE.

[64] Lee J., Shin A., Cho S., Choi J.-Y., Kang D., Lee J.-K. (2020). Marital status and the prevalence of obesity in a Korean population. Obes. Res. Clin. Pract..

[65] Letamo G. (2011). The prevalence of, and factors associated with, overweight and obesity in Botswana. J. Biosoc. Sci..

[66] Liao C., Gao W., Cao W., Lv J., Yu C., Wang S., Pang Z., Cong L., Dong Z., Wu F. (2018). Association of Educational Level and Marital Status with Obesity: A Study of Chinese Twins. Twin Res. Hum. Genet..

[67] Mangemba N.T., Sebastian M.S. (2020). Societal risk factors for overweight and obesity in women in Zimbabwe: A cross-sectional study. BMC Public Health.

[68] Memish Z.A., El Bcheraoui C., Tuffaha M., Robinson M., Daoud F., Jaber S., Mikhitarian S., Al Saeedi M., AlMazroa M.A., Mokdad A.H. (2014). Obesity and Associated Factors—Kingdom of Saudi Arabia, 2013. Prev. Chronic Dis..

[69] Mistretta A., Marventano S., Platania A., Godos J., Galvano F., Grosso G. (2017). Metabolic profile of the Mediterranean healthy Eating, Lifestyle and Aging (MEAL) study cohort. Mediterr. J. Nutr. Metab..

[70] Mkuu R.S., Epnere K., Chowdhury M.A.B. (2018). Prevalence and Predictors of Overweight and Obesity among Kenyan Women. Prev. Chronic Dis..

[71] Modjadji P. (2020). Socio-demographic Determinants of Overweight and Obesity among Mothers of Primary School Children Living in a Rural Health and Demographic Surveillance System Site, South Africa. Open Public Health J..

[72] Moise I.K., Kangmennaang J., Halwiindi H., Grigsby-Toussaint D.S., Fuller D.O. (2019). Increase in Obesity among Women of Reproductive Age in Zambia, 2002–2014. J. Women’s Health.

[73] Mukora-Mutseyekwa F., Zeeb H., Nengomasha L., Adjei N.K. (2019). Trends in Prevalence and Related Risk Factors of Overweight and Obesity among Women of Reproductive Age in Zimbabwe, 2005–2015. Int. J. Environ. Res. Public Health.

[74] Mustafa J., Salle N.M., Isa Z.M., Gha H.F. (2013). Overweight Problem among Primary Health Care Workers in Suburban District of Hulu Langat, Selangor, Malaysia. Pak. J. Nutr..

[75] Najafi F., Pasdar Y., Hamzeh B., Rezaei S., Nazar M.M., Soofi M. (2018). Measuring and Decomposing Socioeconomic Inequalities in Adult Obesity in Western Iran. J. Prev. Med. Public Health.

[76] Oguoma V.M., Coffee N.T., Alsharrah S., Abu-Farha M., Al-Refaei F.H., Al-Mulla F., Daniel M. (2021). Prevalence of overweight and obesity, and associations with socio-demographic factors in Kuwait. BMC Public Health.

[77] Okop K.J., Levitt N., Puoane T. (2015). Factors Associated with Excessive Body Fat in Men and Women: Cross-Sectional Data from Black South Africans Living in a Rural Community and an Urban Township. PLoS ONE.

[78] Oliveira A.J., Rostila M., de Leon A.P., Lopes C.S. (2013). The influence of social relationships on obesity: Sex differences in a longitudinal study. Obesity.

[79] Omar S.M., Taha Z., Hassan A.A., Al-Wutayd O., Adam I. (2020). Prevalence and factors associated with overweight and central obesity among adults in the Eastern Sudan. PLoS ONE.

[80] Ortiz-Moncada R., García M., González-Zapata L.I., Fernandez E., Álvarez-Dardet C. (2010). Incidence of overweight and obesity in a Mediterranean population-based cohort: The Cornellà Health Interview Survey Follow-up Study (CHIS.FU). Prev. Med..

[81] Pereko K.K., Setorglo J., Owusu W.B., Tiweh J.M., Achampong E.K. (2012). Overnutrition and associated factors among adults aged 20 years and above in fishing communities in the urban Cape Coast Metropolis, Ghana. Public Health Nutr.

[82] Puciato D., Rozpara M. (2020). Demographic and Socioeconomic Determinants of Body Mass Index in People of Working Age. Int. J. Environ. Res. Public Health.

[83] Qureshi S.A., Straiton M., Gele A.A. (2020). Associations of socio-demographic factors with adiposity among immigrants in Norway: A secondary data analysis. BMC Public Health.

[84] Wang R., Zhang P., Gao C., Li Z., Lv X., Song Y., Yu Y., Li B. (2016). Prevalence of overweight and obesity and some associated factors among adult residents of northeast China: A cross-sectional study. BMJ Open.

[85] Reynolds S.L., Hagedorn A., Yeom J., Saito Y., Yokoyama E., Crimmins E.M. (2008). A Tale of Two Countries---the United States and Japan: Are Differences in Health Due to Differences in Overweight?. J. Epidemiology.

[86] Tu R., Hou J., Liu X., Li R., Dong X., Pan M., Yin S., Hu K., Mao Z., Huo W. (2020). Low socioeconomic status aggravated associations of exposure to mixture of air pollutants with obesity in rural Chinese adults: A cross-sectional study. Environ. Res..

[87] Santos A.-C., Barros H. (2003). Prevalence and determinants of obesity in an urban sample of Portuguese adults. Public Health.

[88] Sarlio-Lahteenkorva S., Lahelma E. (1999). The association of body mass index with social and economic disadvantage in women and men. Int. J. Epidemiol..

[89] Sartorius B., Veerman L.J., Manyema M., Chola L., Hofman K. (2015). Determinants of Obesity and Associated Population Attributability, South Africa: Empirical Evidence from a National Panel Survey, 2008-2012. PLoS ONE.

[90] Segheto W., Hallal P.C., Marins J.C.B., da Silva D.C.G., Coelho F.A., Ribeiro A.Q., Morais S.H.O., Longo G.Z. (2018). Factors associated with body adiposity index (BAI) in adults: Population-based study. Cienc. Saude Coletiva.

[91] Seubsman S.-A., Lim L.L.-Y., Banwell C., Sripaiboonkit N., Kelly M., Bain C., Sleigh A. (2010). Socioeconomic Status, Sex, and Obesity in a Large National Cohort of 15–87-Year-Old Open University Students in Thailand. J. Epidemiol..

[92] Ahmed S.H., Meyer H.E., Kjøllesdal M.K., Madar A.A. (2018). Prevalence and Predictors of Overweight and Obesity among Somalis in Norway and Somaliland: A Comparative Study. J. Obes..

[93] A Shayo G., Mugusi F.M. (2011). Prevalence of obesity and associated risk factors among adults in Kinondoni municipal district, Dar es Salaam Tanzania. BMC Public Health.

[94] Sidik S.M., Rampal L. (2009). The prevalence and factors associated with obesity among adult women in Selangor, Malaysia. Asia Pac. Fam. Med..

[95] Sim S., Laohasiriwong W. (2019). Fast Food Consumption, Overweight and Obesity among Working Age Persons in Cambodia. J. Clin. Diagn. Res..

[96] Smith T.J., Marriott B.P., Dotson L., Bathalon G.P., Funderburk L., White A., Hadden L., Young A.J. (2012). Overweight and Obesity in Military Personnel: Sociodemographic Predictors. Obesity.

[97] Sobal J., Hanson K.L., Frongillo E.A. (2009). Gender, Ethnicity, Marital Status, and Body Weight in the United States. Obesity.

[98] Song N., Liu F., Han M., Zhao Q., Zhai H., Li X.-M., Du G.-L., Li X.-M., Yang Y.-N. (2019). Prevalence of overweight and obesity and associated risk factors among adult residents of northwest China: A cross-sectional study. BMJ Open.

[99] Suzana S., Kee C.C., Jamaludin A.R., Noor Safiza M.N., Khor G.L., Jamaiyah H., Geeta A., Ahmad Ali Z., Rahmah R., Ruzita A.T. (2012). The Third National Health and Morbidity Survey: Prevalence of obesity, and abdominal obesity among the Malaysian elderly population. Asia Pac. J. Public Health.

[100] Suzuki W., Kuriki K. (2019). Associations between family factors and body weight gain from 20 years old. BMC Women’s Health.

[101] Taghdir M., Alimohamadi Y., Sepandi M., Rezaianzadeh A., Abbaszadeh S., Mahmud F.M. (2020). Association between parity and obesity: A cross sectional study on 6,447 Iranian females. J. Prev. Med. Hyg..

[102] Tanwi T.S., Chakrabarty S., Hasanuzzaman S. (2019). Double burden of malnutrition among ever-married women in Bangladesh: A pooled analysis. BMC Women’s Health.

[103] Tchicaya A., Lorentz N. (2012). Socioeconomic inequality and obesity prevalence trends in luxembourg, 1995–2007. BMC Res. Notes.

[104] Torp J.A., Berggren V., Erlandsson L.-K., Westergren A. (2015). Weight Status among Somali Immigrants in Sweden in Relation to Sociodemographic Characteristics, Dietary Habits and Physical Activity. Open Public Health J..

[105] Tzotzas T., Vlahavas G., Papadopoulou S.K., Kapantais E., Kaklamanou D., Hassapidou M. (2010). Marital status and educational level associated to obesity in Greek adults: Data from the National Epidemiological Survey. BMC Public Health.

[106] Gigante D.P., Moura E.C.D., Sardinha L.M.V. (2009). Prevalence of overweight and obesity and associated factors. Rev. Saude Publica.

[107] Veghari G., Sedaghat M., Joshaghani H., Hoseini A., Niknezhad F., Angizeh A., Tazik E., Moharloei P. (2010). The Prevalence of Obesity and its Related Risk Factor in the North of Iran in 2006 ARTICLE INFORMATION ABSTRACT. J. Res. Health Sci. (JRHS).

[108] Wardle J., Waller J., Jarvis M.J. (2002). Sex Differences in the Association of Socioeconomic Status with Obesity. Am. J. Public Health.

[109] Watson K., Roberts B., Chow C., Goryakin Y., Rotman D., Gasparishvili A., Haerpfer C., McKee M. (2012). Micro- and meso-level influences on obesity in the former Soviet Union: A multi-level analysis. Eur. J. Public Health.

[110] Weiderpass E., Botteri E., Longenecker J.C., Alkandari A., Al-Wotayan R., Al Duwairi Q., Tuomilehto J. (2019). The Prevalence of Overweight and Obesity in an Adult Kuwaiti Population in 2014. Front. Endocrinol..

[111] Woo J., Leung S., Ho S., Sham A., Lam T., Janus E. (1999). Influence of educational level and marital status on dietary intake, obesity and other cardiovascular risk factors in a Hong Kong Chinese population. Eur. J. Clin. Nutr..

[112] Liu X., Wu W., Mao Z., Huo W., Tu R., Qian X., Zhang X., Tian Z., Zhang H., Jiang J. (2018). Prevalence and influencing factors of overweight and obesity in a Chinese rural population: The Henan Rural Cohort Study. Sci. Rep..

[113] Wang M., Xu S., Liu W., Zhang C., Zhang X., Wang L., Liu J., Zhu Z., Hu J., Luo X. (2020). Prevalence and changes of BMI categories in China and related chronic diseases: Cross-sectional National Health Service Surveys (NHSSs) from 2013 to 2018. EClinicalMedicine.

[114] Yu S., Xing L., Du Z., Tian Y., Jing L., Yan H., Lin M., Zhang B., Liu S., Pan Y. (2019). Prevalence of Obesity and Associated Risk Factors and Cardiometabolic Comorbidities in Rural Northeast China. BioMed Res. Int..

[115] Zapata M.E., Bibiloni M.d.M., Tur J.A. (2016). Prevalence of overweihgt, obesity, abdominal-obesity and short stature of adult population of Rosario, Argentina. Nutr. Hosp..

[116] Zhang L., Wang Z., Wang X., Chen Z., Shao L., Tian Y., Zheng C., Li S., Zhu M., Gao R. (2020). Prevalence of overweight and obesity in China: Results from a cross-sectional study of 441 thousand adults, 2012–2015. Obes. Res. Clin. Pract..

[117] Zhou L., Cao D., Si Y., Zhu X., Du L., Zhang Y., Zhou Z. (2020). Income-related inequities of adult obesity and central obesity in China: Evidence from the China Health and Nutrition Survey 1997-2011. BMJ Open.

[118] Jeffery R.W., Rick A.M. (2002). Cross-Sectional and Longitudinal Associations between Body Mass Index and Marriage-Related Factors. Obes. Res..

[119] Marks D.F. (2015). Homeostatic theory of obesity. Health Psychol. Open.

[120] Meltzer A.A., Everhart J.E. (1995). Self-Reported Substantial 1-Year Weight Change among Men and Women in the United States. Obes. Res..

[121] Bove C.F., Sobal J. (2011). Body weight relationships in early marriage. Weight relevance, weight comparisons, and weight talk. Appetite.

